# Supplementary material for: A Likelihood Approach for Real-Time Calibration of Stochastic Compartmental Epidemic Models
Source: PLoS Comput Biol. 2017 Jan 17;13(1):e1005257. doi: 10.1371/journal.pcbi.1005257 (PMC5240920; doi:10.1371/journal.pcbi.1005257)
Supplement: S1 File — (TAR.GZ) [file pcbi.1005257.s014.tar.gz › HSPH_Online-SI-Revision/Additions/PF09/n100-extreme_noisy/n100-extreme_noisy_PF2_table0.pdf]

1	newly 3 weeks spec	7368.61	6810.27	{6692., 6919.}	7.57733	0.0342632	newly 3 weeks cumul	27409.9	20182.5	{19940., 20381.}	26.3678	0.132949	newly 5 weeks spec	4368.49	5845.8	{5712., 5977.}	33.8173	0.126444	newly 5 weeks cumul	37692.1	32461.9	{32177., 32707.}	13.876	0.0648867	newly 54 weeks cumul	51622.1	60729.2	{60070., 61234.}	17.6418	0.0705159
2	newly 3 weeks spec	5965.	6309.34	{6201., 6418.}	5.77267	0.0243363	newly 3 weeks cumul	20994.	20470.5	{20258., 20651.}	2.49362	0.0109781	newly 5 weeks spec	3796.	4810.74	{4704., 4905.}	26.7318	0.102836	newly 5 weeks cumul	29744.	30858.3	{30591., 31086.}	3.74644	0.0159655	newly 50 weeks cumul	42279.	52316.4	{51983., 52581.}	23.7409	0.0925095
3	newly 3 weeks spec	6513.	7856.16	{7721., 8000.}	20.6228	0.0813953	newly 3 weeks cumul	27002.	27601.4	{27438., 27750.}	2.21972	0.00952948	newly 5 weeks spec	3181.	5078.01	{4986., 5163.}	59.6356	0.203085	newly 5 weeks cumul	34839.	39085.4	{38911., 39261.}	12.1886	0.049945	newly 55 weeks cumul	41209.	55677.7	{55528., 55833.}	35.1105	0.130687
4	newly 3 weeks spec	6774.	10152.8	{10025., 10254.}	49.8784	0.175717	newly 3 weeks cumul	33063.	32920.2	{32706., 33148.}	0.570577	0.00248672	newly 5 weeks spec	2428.	6934.21	{6840., 7054.}	185.593	0.455719	newly 5 weeks cumul	39680.	48420.1	{48123., 48687.}	22.0264	0.0864492	newly 59 weeks cumul	42886.	71207.5	{70922., 71448.}	66.0389	0.220208
5	newly 3 weeks spec	8431.	9054.86	{8949., 9161.}	7.3996	0.0309789	newly 3 weeks cumul	31964.	29295.4	{29084., 29509.}	8.3488	0.037874	newly 5 weeks spec	4440.	6376.46	{6262., 6485.}	43.614	0.157152	newly 5 weeks cumul	42439.	43440.9	{43194., 43754.}	2.36075	0.0101256	newly 56 weeks cumul	51326.	65794.8	{65602., 66011.}	28.1899	0.107851
6	newly 3 weeks spec	7676.	7748.73	{7625., 7880.}	1.35735	0.00585082	newly 3 weeks cumul	28666.	23368.8	{23168., 23546.}	18.4792	0.0887398	newly 5 weeks spec	4357.	6161.7	{6068., 6256.}	41.4207	0.150481	newly 5 weeks cumul	38952.	36596.3	{36327., 36791.}	6.04783	0.0270988	newly 54 weeks cumul	50195.	61379.7	{61122., 61642.}	22.2825	0.0873622
7	newly 3 weeks spec	7326.	6567.	{6467., 6674.}	10.3604	0.0475329	newly 3 weeks cumul	27951.	18788.1	{18603., 18969.}	32.7822	0.17253	newly 5 weeks spec	3953.	6085.49	{5942., 6201.}	53.9461	0.187317	newly 5 weeks cumul	37123.	31334.7	{31049., 31642.}	15.5922	0.0736279	newly 52 weeks cumul	46099.	65740.1	{65392., 66031.}	42.6064	0.154136
8	newly 3 weeks spec	7809.	7514.65	{7398., 7636.}	3.76937	0.016715	newly 3 weeks cumul	28877.	22714.8	{22564., 22883.}	21.3396	0.104251	newly 5 weeks spec	4404.	6026.21	{5902., 6139.}	36.8349	0.136144	newly 5 weeks cumul	39237.	35604.2	{35349., 35899.}	9.25863	0.0422015	newly 54 weeks cumul	50550.	60255.1	{59926., 60534.}	19.199	0.0762692
9	newly 3 weeks spec	8491.	7452.1	{7313., 7566.}	12.2353	0.0567182	newly 3 weeks cumul	32010.	21470.1	{21315., 21632.}	32.9269	0.17346	newly 5 weeks spec	4649.	6492.15	{6373., 6611.}	39.6462	0.144986	newly 5 weeks cumul	43099.	35095.8	{34878., 35331.}	18.5694	0.0892179	newly 54 weeks cumul	54004.	64824.	{64544., 65087.}	20.0356	0.0793079
10	newly 3 weeks spec	3970.	9601.58	{9472., 9737.}	141.853	0.383528	newly 3 weeks cumul	22883.	32135.5	{31962., 32338.}	40.4339	0.147468	newly 5 weeks spec	1101.	6366.64	{6240., 6470.}	478.26	0.762082	newly 5 weeks cumul	26005.	46446.9	{46200., 46659.}	78.6074	0.251897	newly 58 weeks cumul	27241.	67037.6	{66830., 67237.}	146.091	0.391094
11	newly 3 weeks spec	6532.	6801.28	{6700., 6910.}	4.12247	0.0175137	newly 3 weeks cumul	23257.	22434.1	{22275., 22591.}	3.53816	0.0156511	newly 5 weeks spec	3974.	4892.15	{4770., 5009.}	23.1039	0.0902074	newly 5 weeks cumul	32341.	33178.	{32931., 33390.}	2.58811	0.0110917	newly 52 weeks cumul	43101.	51809.2	{51452., 52148.}	20.2042	0.0799146
12	newly 3 weeks spec	8024.	7088.38	{6978., 7207.}	11.6603	0.0538801	newly 3 weeks cumul	31244.	20020.8	{19825., 20211.}	35.9212	0.193297	newly 5 weeks spec	4154.	6550.57	{6429., 6651.}	57.6931	0.197779	newly 5 weeks cumul	41376.	33550.5	{33263., 33805.}	18.9132	0.0910573	newly 53 weeks cumul	50863.	68674.9	{68398., 68950.}	35.0193	0.130394
13	newly 3 weeks spec	7496.	5958.47	{5863., 6052.}	20.5113	0.0997268	newly 3 weeks cumul	24982.	17631.9	{17454., 17795.}	29.4217	0.15134	newly 5 weeks spec	4998.	5249.83	{5147., 5336.}	5.03862	0.0212971	newly 5 weeks cumul	36270.	28580.6	{28359., 28823.}	21.2005	0.103484	newly 51 weeks cumul	54152.	57542.2	{57206., 57875.}	6.26056	0.0263682
14	newly 3 weeks spec	5024.	6974.9	{6827., 7102.}	38.8316	0.142437	newly 3 weeks cumul	30056.	17610.4	{17336., 17831.}	41.4082	0.232189	newly 5 weeks spec	1153.	7672.02	{7536., 7793.}	565.396	0.823041	newly 5 weeks cumul	33840.	32862.4	{32419., 33219.}	2.88892	0.0127503	newly 56 weeks cumul	34818.	76696.3	{76418., 76961.}	120.278	0.342968
15	newly 3 weeks spec	5483.	8911.16	{8758., 9042.}	62.5234	0.21088	newly 3 weeks cumul	26035.	29303.7	{28969., 29705.}	12.5551	0.0513427	newly 5 weeks spec	1932.	6130.64	{6004., 6234.}	217.321	0.501458	newly 5 weeks cumul	31301.	42966.9	{42540., 43496.}	37.2701	0.137558	newly 56 weeks cumul	33894.	64033.1	{63537., 64600.}	88.9216	0.276271
16	newly 3 weeks spec	6616.	9744.03	{9640., 9867.}	47.2798	0.168122	newly 3 weeks cumul	30832.	31029.3	{30846., 31228.}	0.696679	0.00301232	newly 5 weeks spec	2584.	6858.77	{6741., 6963.}	165.432	0.423916	newly 5 weeks cumul	37486.	46252.8	{45989., 46474.}	23.3868	0.0912656	newly 56 weeks cumul	41249.	69738.6	{69613., 69851.}	69.0673	0.228059
17	newly 3 weeks spec	6952.	6754.97	{6652., 6862.}	2.83674	0.012532	newly 3 weeks cumul	24739.	21284.7	{21118., 21418.}	13.9629	0.0653209	newly 5 weeks spec	4470.	5312.53	{5207., 5406.}	18.8485	0.0749476	newly 5 weeks cumul	34818.	32677.7	{32509., 32868.}	6.14719	0.0275568	newly 51 weeks cumul	48456.	56864.6	{56579., 57100.}	17.3531	0.0694916
18	newly 3 weeks spec	7355.	5230.88	{5137., 5352.}	28.8799	0.148061	newly 3 weeks cumul	26994.	14515.1	{14350., 14672.}	46.2284	0.269463	newly 5 weeks spec	4020.	5244.42	{5133., 5351.}	30.4582	0.115409	newly 5 weeks cumul	36463.	25100.5	{24845., 25309.}	31.1617	0.162181	newly 52 weeks cumul	45100.	61308.6	{61044., 61603.}	35.9393	0.133342
19	newly 3 weeks spec	8310.	7171.6	{7038., 7285.}	13.6992	0.0640217	newly 3 weeks cumul	31919.	20176.	{19995., 20377.}	36.7901	0.199229	newly 5 weeks spec	4512.	6644.74	{6518., 6748.}	47.2682	0.168068	newly 5 weeks cumul	42585.	33890.1	{33605., 34139.}	20.4177	0.0991952	newly 54 weeks cumul	52753.	69409.3	{69112., 69730.}	31.5741	0.119168
20	newly 3 weeks spec	6547.	11124.7	{10954., 11245.}	69.9204	0.230222	newly 3 weeks cumul	38691.	31131.5	{30919., 31330.}	19.5381	0.094416	newly 5 weeks spec	1542.	8852.88	{8716., 8980.}	474.117	0.758973	newly 5 weeks cumul	43499.	50268.2	{50006., 50526.}	15.5616	0.0628105	newly 59 weeks cumul	44967.	82161.2	{81976., 82325.}	82.7144	0.261772
21	newly 3 weeks spec	7119.	8934.06	{8838., 9047.}	25.496	0.0986082	newly 3 weeks cumul	29356.	29479.7	{29314., 29624.}	0.486544	0.00210549	newly 5 weeks spec	3172.	6106.49	{5995., 6214.}	92.5123	0.284422	newly 5 weeks cumul	37313.	43106.8	{42937., 43304.}	15.5275	0.0626831	newly 56 weeks cumul	42719.	63825.3	{63677., 63963.}	49.4073	0.17437
22	newly 3 weeks spec	6759.	9185.39	{9067., 9335.}	35.8987	0.133187	newly 3 weeks cumul	29716.	31504.3	{31293., 31716.}	6.0178	0.025373	newly 5 weeks spec	2827.	5964.96	{5850., 6085.}	111.	0.324234	newly 5 weeks cumul	37047.	44980.2	{44724., 45275.}	21.414	0.0842639	newly 57 weeks cumul	41620.	64077.6	{63794., 64492.}	53.9587	0.187401
23	newly 3 weeks spec	6270.	5340.73	{5235., 5431.}	14.8209	0.0697142	newly 3 weeks cumul	22584.	15504.3	{15381., 15634.}	31.3481	0.163357	newly 5 weeks spec	3912.	5006.18	{4910., 5112.}	27.9698	0.107049	newly 5 weeks cumul	31596.	25762.1	{25519., 25962.}	18.464	0.0886594	newly 50 weeks cumul	43028.	58460.8	{58148., 58745.}	35.8669	0.13311
24	newly 3 weeks spec	7567.	5272.86	{5169., 5386.}	30.3177	0.156931	newly 3 weeks cumul	27322.	15144.2	{14975., 15289.}	44.5715	0.256281	newly 5 weeks spec	4888.	5047.62	{4939., 5151.}	3.30442	0.0140607	newly 5 weeks cumul	38440.	25449.8	{25218., 25698.}	33.7935	0.17911	newly 50 weeks cumul	52801.	59593.5	{59291., 59926.}	12.8642	0.052553
25	newly 3 weeks spec	6782.	6132.91	{5988., 6251.}	9.57078	0.0437458	newly 3 weeks cumul	30207.	16553.2	{16326., 16709.}	45.2008	0.261242	newly 5 weeks spec	2743.	6271.71	{6148., 6406.}	128.644	0.359103	newly 5 weeks cumul	37399.	29170.9	{28800., 29477.}	22.0009	0.107926	newly 53 weeks cumul	41988.	69942.2	{69554., 70332.}	66.5765	0.22161
26	newly 3 weeks spec	7069.	6828.94	{6733., 6928.}	3.39595	0.0150336	newly 3 weeks cumul	23874.	22012.1	{21848., 22168.}	7.79874	0.0352709	newly 5 weeks spec	4701.	5171.09	{5055., 5281.}	9.99979	0.0413325	newly 5 weeks cumul	34401.	33204.2	{32982., 33410.}	3.47888	0.0153829	newly 51 weeks cumul	50936.	55440.2	{55151., 55695.}	8.84286	0.0367963
27	newly 3 weeks spec	6335.	6412.23	{6299., 6507.}	1.51081	0.00650459	newly 3 weeks cumul	22631.	21315.5	{21165., 21504.}	5.81282	0.0260157	newly 5 weeks spec	3937.	4550.79	{4444., 4652.}	15.5903	0.0628491	newly 5 weeks cumul	31837.	31350.7	{31136., 31579.}	1.52763	0.00669296	newly 52 weeks cumul	45079.	48540.4	{48137., 48875.}	7.67854	0.0321155
28	newly 3 weeks spec	7243.	7768.95	{7631., 7894.}	7.26149	0.0304091	newly 3 weeks cumul	27640.	27778.7	{27540., 28065.}	0.674711	0.00291615	newly 5 weeks spec	4186.	4905.47	{4789., 5009.}	17.1875	0.0688251	newly 5 weeks cumul	37414.	38926.4	{38640., 39294.}	4.04244	0.0172016	newly 56 weeks cumul	48001.	54534.3	{54122., 55064.}	13.6108	0.0554101
29	newly 3 weeks spec	6769.	5509.47	{5419., 5613.}	18.6073	0.0894598	newly 3 weeks cumul	23489.	15960.1	{15821., 16102.}	32.0527	0.167839	newly 5 weeks spec	4398.	5157.78	{5046., 5269.}	17.2756	0.0691485	newly 5 weeks cumul	33395.	26544.9	{26329., 26767.}	20.5123	0.0997091	newly 50 weeks cumul	46315.	59554.5	{59237., 59872.}	28.5858	0.109189
30	newly 3 weeks spec	8192.	10018.	{9868., 10147.}	22.2905	0.0873723	newly 3 weeks cumul	34290.	32313.2	{32177., 32458.}	5.76498	0.0257903	newly 5 weeks spec	3775.	6904.81	{6803., 7011.}	82.9089	0.262203	newly 5 weeks cumul	43617.	47719.7	{47549., 47882.}	9.40613	0.0390399	newly 59 weeks cumul	50418.	70611.6	{70452., 70755.}	40.0524	0.14629
31	newly 3 weeks spec	7625.	10948.4	{10817., 11091.}	43.585	0.157085	newly 3 weeks cumul	37142.	33275.5	{33031., 33492.}	10.41	0.0477466	newly 5 weeks spec	2494.	7927.34	{7811., 8035.}	217.856	0.502203	newly 5 weeks cumul	43977.	50786.5	{50445., 51038.}	15.4841	0.0625188	newly 60 weeks cumul	46875.	77765.1	{77490., 78010.}	65.8989	0.219842
32	newly 3 weeks spec	6240.	8786.52	{8663., 8890.}	40.8096	0.148605	newly 3 weeks cumul	27473.	30193.5	{30009., 30378.}	9.90238	0.0410018	newly 5 weeks spec	2353.	5737.35	{5636., 5837.}	143.831	0.387043	newly 5 weeks cumul	33807.	43117.4	{42907., 43320.}	27.5399	0.10564	newly 57 weeks cumul	37286.	61613.9	{61407., 61831.}	65.2468	0.218126
33	newly 3 weeks spec	8467.	8403.22	{8280., 8545.}	1.1598	0.00506907	newly 3 weeks cumul	28949.	28468.7	{28154., 28723.}	1.65916	0.0072803	newly 5 weeks spec	5209.	5646.64	{5510., 5797.}	8.40161	0.0349478	newly 5 weeks cumul	40750.	41107.8	{40763., 41518.}	0.978086	0.00422221	newly 55 weeks cumul	56254.	60184.5	{59583., 61132.}	6.98702	0.0293067
34	newly 3 weeks spec	6053.	6064.42	{5925., 6189.}	1.30481	0.00566113	newly 3 weeks cumul	19976.	18835.2	{18527., 19045.}	5.71085	0.0255612	newly 5 weeks spec	4286.	4919.72	{4825., 5013.}	14.7858	0.0598431	newly 5 weeks cumul	29425.	29310.	{28967., 29630.}	0.721733	0.00314743	newly 50 weeks cumul	43351.	53337.5	{52964., 53668.}	23.0363	0.0900271
35	newly 3 weeks spec	8823.	8044.69	{7921., 8168.}	8.82138	0.0401344	newly 3 weeks cumul	31843.	23440.8	{23249., 23587.}	26.3862	0.133048	newly 5 weeks spec	5272.	6689.14	{6593., 6807.}	26.8805	0.103355	newly 5 weeks cumul	44053.	37670.8	{37457., 37897.}	14.4875	0.0679765	newly 55 weeks cumul	57367.	64668.1	{64392., 64905.}	12.727	0.0520255
36	newly 3 weeks spec	6496.	6841.53	{6702., 6974.}	5.31912	0.0224547	newly 3 weeks cumul	23327.	22802.3	{22527., 23067.}	2.24924	0.00989869	newly 5 weeks spec	4189.	4768.3	{4660., 4881.}	13.8291	0.0561711	newly 5 weeks cumul	32804.	33361.7	{33012., 33650.}	1.71284	0.00736391	newly 53 weeks cumul	44915.	50149.4	{49508., 50726.}	11.654	0.0478568
37	newly 3 weeks spec	6929.	9526.96	{9400., 9649.}	37.494	0.138261	newly 3 weeks cumul	31730.	31554.	{31379., 31712.}	0.593476	0.00258793	newly 5 weeks spec	2540.	6394.08	{6302., 6499.}	151.735	0.400912	newly 5 weeks cumul	38553.	45897.1	{45672., 46096.}	19.0493	0.0757241	newly 58 weeks cumul	42395.	66909.2	{66747., 67125.}	57.8232	0.19817
38	newly 3 weeks spec	5419.	10836.7	{10698., 10985.}	99.9766	0.300957	newly 3 weeks cumul	32641.	31893.8	{31666., 32124.}	2.28915	0.0100632	newly 5 weeks spec	1229.	8188.54	{8063., 8297.}	566.277	0.823627	newly 5 weeks cumul	36650.	49806.8	{49578., 50009.}	35.8984	0.133212	newly 58 weeks cumul	37768.	78536.4	{78347., 78720.}	107.944	0.317946
39	newly 3 weeks spec	5970.	7533.54	{7420., 7650.}	26.1899	0.10099	newly 3 weeks cumul	21972.	23650.6	{23355., 23920.}	7.63981	0.0319538	newly 5 weeks spec	3152.	5863.59	{5752., 5976.}	86.0276	0.269527	newly 5 weeks cumul	29579.	36277.3	{35852., 36643.}	22.6453	0.0886374	newly 52 weeks cumul	36448.	61293.8	{60939., 61584.}	68.1679	0.22574
40	newly 3 weeks spec	6214.	7410.49	{7295., 7536.}	19.2547	0.0764374	newly 3 weeks cumul	22734.	25434.6	{25049., 25799.}	11.8791	0.0487228	newly 5 weeks spec	3630.	4942.11	{4824., 5051.}	36.1463	0.133944	newly 5 weeks cumul	31216.	36509.3	{36123., 36921.}	16.9569	0.0680095	newly 53 weeks cumul	41146.	52964.1	{52349., 53463.}	28.7224	0.109638
41	newly 3 weeks spec	5376.	7557.83	{7227., 7885.}	40.5846	0.147712	newly 3 weeks cumul	21206.	26131.4	{25355., 27026.}	23.2264	0.0905677	newly 5 weeks spec	2830.	4906.82	{4538., 5313.}	73.3859	0.238174	newly 5 weeks cumul	27896.	37216.2	{35746., 38676.}	33.4105	0.124997	newly 54 weeks cumul	33607.	52814.3	{49045., 57080.}	57.1527	0.195436
42	newly 3 weeks spec	4668.	9554.59	{9446., 9675.}	104.683	0.311058	newly 3 weeks cumul	24350.	31831.5	{31650., 32029.}	30.7248	0.116353	newly 5 weeks spec	1451.	6386.8	{6284., 6501.}	340.165	0.643581	newly 5 weeks cumul	28470.	46172.4	{45971., 46370.}	62.1791	0.209992	newly 58 weeks cumul	30111.	66983.9	{66809., 67104.}	122.456	0.347244
43	newly 3 weeks spec	8069.	4666.11	{4564., 4765.}	42.1724	0.237927	newly 3 weeks cumul	29202.	12671.	{12514., 12796.}	56.6092	0.362619	newly 5 weeks spec	4750.	5005.96	{4875., 5113.}	5.38863	0.0227153	newly 5 weeks cumul	40208.	22603.4	{22388., 22813.}	43.7838	0.250153	newly 51 weeks cumul	53458.	64302.1	{63889., 64718.}	20.2853	0.0802069
44	newly 3 weeks spec	6354.	8691.77	{8573., 8800.}	36.7921	0.136035	newly 3 weeks cumul	27932.	29936.	{29774., 30102.}	7.1746	0.0300881	newly 5 weeks spec	2650.	5648.04	{5542., 5759.}	113.134	0.328609	newly 5 weeks cumul	34711.	42688.	{42510., 42856.}	22.9811	0.0898362	newly 57 weeks cumul	39102.	60974.7	{60815., 61137.}	55.9375	0.19295
45	newly 3 weeks spec	4797.	9661.17	{9529., 9787.}	101.4	0.304038	newly 3 weeks cumul	26104.	30851.6	{30664., 31027.}	18.1874	0.0725663	newly 5 weeks spec	1420.	6795.83	{6700., 6904.}	378.58	0.679923	newly 5 weeks cumul	30202.	45932.4	{45746., 46103.}	52.0839	0.182081	newly 56 weeks cumul	31840.	69182.5	{69030., 69334.}	117.282	0.337023
46	newly 3 weeks spec	8356.	9618.87	{9494., 9726.}	15.1133	0.0611079	newly 3 weeks cumul	33903.	29938.2	{29760., 30135.}	11.6945	0.0540177	newly 5 weeks spec	3539.	7027.44	{6889., 7145.}	98.5713	0.297876	newly 5 weeks cumul	42756.	45397.	{45228., 45583.}	6.17689	0.026027	newly 56 weeks cumul	48435.	70522.6	{70334., 70680.}	45.6026	0.163168
47	newly 3 weeks spec	6682.	9502.02	{9370., 9623.}	42.2032	0.152883	newly 3 weeks cumul	30081.	31714.1	{31474., 31968.}	5.42898	0.0229515	newly 5 weeks spec	2681.	6318.63	{6208., 6430.}	135.682	0.372287	newly 5 weeks cumul	37114.	45940.8	{45687., 46217.}	23.783	0.0926556	newly 58 weeks cumul	40818.	66581.8	{66290., 66870.}	63.1187	0.212501
48	newly 3 weeks spec	8702.	10628.9	{10510., 10736.}	22.1435	0.0868509	newly 3 weeks cumul	37680.	33368.9	{33141., 33583.}	11.4415	0.0527747	newly 5 weeks spec	3585.	7477.43	{7374., 7591.}	108.575	0.319236	newly 5 weeks cumul	46811.	49996.8	{49766., 50211.}	6.80556	0.0285903	newly 58 weeks cumul	52174.	74938.3	{74704., 75142.}	43.6315	0.157249
49	newly 3 weeks spec	4127.	11030.6	{10880., 11178.}	167.278	0.426938	newly 3 weeks cumul	27465.	33109.8	{32885., 33359.}	20.5527	0.0811704	newly 5 weeks spec	921.	8076.55	{7958., 8187.}	776.933	0.942937	newly 5 weeks cumul	30359.	50894.5	{50551., 51197.}	67.6421	0.224378	newly 60 weeks cumul	31206.	78425.7	{78095., 78663.}	151.316	0.400219
50	newly 3 weeks spec	6476.	6553.38	{6451., 6644.}	1.41847	0.00610429	newly 3 weeks cumul	23838.	20254.3	{20122., 20412.}	15.0338	0.0707602	newly 5 weeks spec	3992.	5350.12	{5238., 5458.}	34.021	0.127126	newly 5 weeks cumul	32964.	31634.	{31405., 31809.}	4.03464	0.017891	newly 51 weeks cumul	44879.	57663.5	{57425., 57914.}	28.4865	0.108855
